# Participants’ awareness of ethical compliance, safety and protection during participation in pharmaceutical industry clinical trials: a controlled survey

**DOI:** 10.1186/s12910-018-0344-8

**Published:** 2019-01-08

**Authors:** Gerardo González-Saldivar, René Rodríguez-Gutiérrez, Jose Luis Viramontes-Madrid, Alejandro Salcido-Montenegro, Neri Alejandro Álvarez-Villalobos, Victoria González-Nava, José Gerardo González-González

**Affiliations:** 1 Ophthalmology Division, Fundación Hospital Nuestra Señora de la Luz, IAP, Ciudad de México, Mexico; 2 Endocrinology Division, Department of Internal Medicine, “Dr. José E. González” University Hospital, Universidad Autonoma de Nuevo Leon, 64460 Monterrey, Mexico; 3 Knowledge and Evaluation Research Unit in Endocrinology, Mayo Clinic, Rochester, MN 55905 USA; 4 Division of Endocrinology, Diabetes, Metabolism and Nutrition, Department of Medicine, Mayo Clinic, Rochester, MN 55905 USA; 5 Instituto Nacional de Salud Pública, Cuernavaca, Morelos Mexico; 6 Clinical Research Unit, “Dr. José E. González” University Hospital, Universidad Autonoma de Nuevo Leon, 64460 Monterrey, Mexico; 7 Servicio de Endocrinología, Departamento de Medicina Interna, Hospital Universitario “Dr. José E. González”, Universidad Autonoma de Nuevo Leon, Av. Francisco I. Madero y Av. Gonzalitos s/n, Colonia Mitras Centro, CP. 64460 Monterrey, Nuevo León Mexico

**Keywords:** Good clinical practices, Perception, Clinical trials, Pharmaceutical industry, Ethics committees

## Abstract

**Background:**

The rapid increase of industry-sponsored clinical research towards developing countries has led to potentially complex ethical issues to assess. There is scarce evidence about the perception of these participants about the ethical compliance, security, and protection. We sought to evaluate and contrast the awareness and perception of participants and non-participants of industry-sponsored research trials (ISRT) on ethical, safety, and protection topics.

**Methods:**

A Cases-control survey conducted at twelve research sites in México. Previous and current participants of ISRT (cases) as well as non-participants (controls) with one of four chronic diseases, were asked to complete the survey which focused on ethical compliance and protection issues of ISRT, and the perception of participating in a trial.

**Results:**

A total of 604 cases and 604 controls were surveyed. Cases significantly answered that ethics committees are aware of what is happening in studies (50.5% vs. 33.8%, *P* = ≤ 0.001), and that medical care of industry-sponsored research trials is better than their usual medical care (77.2% vs. 38.2%, *P* = < 0.001). The same proportion of cases and controls thought patients must receive economical reimbursement for participating in a research study (49.5% vs. 53.1%, *P* = 0.205). The informed consent of the pharmaceutical clinical trial was fully read by 90.4% of the cases. Most cases were satisfied or very satisfied with their overall study participation (35.6 and 62.3%, respectively).

**Conclusion:**

Previous and current participants of industry-sponsored research trials have a more positive attitude towards ethics committees, the quality of medical care of the research trials, and the main purpose of economical reimbursements, when compared to non-participants.

## Background

The major participation and transfer of pharmaceutical industry clinical trials from developed to developing countries has occurred over the last three decades [1–3]. In the last 10 years, clinical trial registry in low- and low-middle income countries has increased around 260% [4, 5]. Currently, one in four industry-sponsored research trials (ISRT) include participants outside of high-income countries [6, 7]. Furthermore, clinical research protocols conducted in developing countries frequently have a proportional major participation in recruitment than developed countries [2, 6]. There are many reasons to explain the progressive growth of ISRT in developing countries: a significantly lower cost for execution, the growth of the pharmaceutical market in some of these regions, and the large populations interested in protocol participation as an alternative for their health care.

The rapid increase of ISRT in developing countries has led to potential complex ethical issues and cases of researcher misconduct [1]. Participants have an overall good acceptance of ISRT, frequently identifying them as a satisfactory way to receive high-quality medical care [8]. Widespread poverty, lack of education and very limited access to good-quality health services in many developing countries make the ethical conduction process of these protocols uncertain, particularly during recruitment and informed consent (IC) phases [1]. Additionally, undue incentives and potential local institutional review boards without a highly professional performance are other factors. Even though some studies have assessed the opinion of experts regarding these topics, there is a paucity of evidence concerning the participant’s perception [9–11].

We carried out a multicenter, disease-paired controlled survey in México, in academic and non-academic pharmaceutical clinical research sites. Our hypothesis was that previous and current participants of research trails would have different perspective and opinions about clinical trials when compared to non-participants. The primary endpoint was to contrast the awareness and perception of participants and non-participants of the ISRT on a wide variety of ethical, safety and protection topics in a large population with one out of four highly prevalent chronic diseases: type 2 diabetes (T2DM), arterial hypertension (HT), chronic obstructive pulmonary disease (COPD), and rheumatoid arthritis (RA). Secondary endpoints were to contrast their perception by gender, age, disease, and research site.

## Methods

### Design and validation of the survey

We report the second section of a multicenter survey on clinical trial perceptions, which focused on ethical compliance and protection issues of ISRT, along with the overall perception of participating in a trial. Rationale, design, and detailed validation of the survey have been previously published [8]. Validation of the survey process consisted of a two-phase pre-pilot study and a pilot study. The original draft was tested in two groups of 7–8 participants each (80% of which were previously involved in a clinical trial). Twelve individual semi-structured interviews were conducted at 6 out of 12 research sites (2 interviews per site). In order to cover the four main diseases (T2DM, HAS, COPD, RA) that our survey focused on in our pilot study, these 6 centers were randomly selected based on their specialty and the clinical trials that they were conducting. Questions were revised and modified considering key points mentioned by participants in each stage. Thereafter, the modified questionnaire was applied to 30 individuals who fulfilled the same eligibility criteria. Due to the amount of information, the diversity of issues, and for better communication to readers, the results of the survey were divided into two documents.

### Study participants and research sites

Institutional review board (IRB) approval was obtained. Comité de Ética en Investigación del Hospital Universitario “Dr Jose Eleuterio González”, CONBIOETICA -19-CEI-001-20,160,404. All participants’ sites were approved by our IRB before participating in the recruitment of the study. All participants provided written informed consent. Men and women, aged 18 to 80 years, who have lived in Mexico the last 10 years and were diagnosed with T2DM, HT, COPD, or RA, were eligible for participation. Cases were former or current participants of phase II or III pharmaceutical clinical trials who had attended at least their sixth visit. If participants had more than one of the diseases included in this study, they were assigned to the disease subgroup that matched with the clinical trial. Disease-matching controls had never participated or been invited to participate in pharmaceutical clinical trials. They were recruited while attending the primary care or specialty outpatient clinics of the clinical research sites. All participants answered the self-survey independently. The surveys were applied at 12 research sites in México. Each facility had a professional pharmaceutical research team and had been conducting pharmaceutical research studies for at least 10 years. All research sites also offered primary care and specialty outpatient clinic services. It is noteworthy that more than 75% of the population have access to health care, while the rest of them have access public health system of national coverage.

### Study protocol and procedures

Cases were recruited while attending their research study site and controls at the outpatient clinics. Both cases and controls were recruited in a consecutive manner while attending their research study site and at the outpatient clinics, respectively. One hundred surveys were applied in each research site: 50 for cases and 50 for controls. A staff member, not involved in the participants’ clinical research, obtained the participant’s demographic data, applied the survey, clarified any question and ensured survey completion. All surveys were sent to a central site for data management and analysis. Cases and controls answered 9 multiple-choice questions, each with 2 to 9 response options. The cases group answered 12 additional questions focused on their satisfaction and experience while participating in a research clinical trial. Neither cases nor did controls receive economical reimbursement for their participation in our survey study. Our study was conducted separately and independently of any pharmaceutical clinical trials that cases were taking part at the moment of the survey application.

### Statistical analysis

Statistical analyses were done using IBM SPSS Statistics 20.0 (IBM Corporation, Armonk, NY). Continuous variables are expressed as means ± standard deviation; categorical variables are expressed as frequencies and percentages. Categorical variables were compared using the Chi-square test and the Fisher exact test for 2 × 2 tables. For quantitative comparative variables, Student’s *t* test or the Mann Whitney U test were employed; distribution of numerical variables was confirmed by the Kolmogorov-Smirnov test. A *P* value ≤0.05 was considered statistically significant.

## Results

### Study population

The demographic and clinical characteristics of the study population are shown in Table 1. Every patient approached agreed to answer the survey. A total of 604 cases and 604 controls participated. Except for mean age (cases were older than controls, 54.9 ± 13.9 vs. 47.1 ± 15.5 years, respectively, *P* ≤ 0.001), there was no other statistically significant difference in the demographic variables. Female to male ratio in both groups was 2:1. Cases who were participating in a pharmaceutical clinical trial for the first time accounted for 65.4% of the whole group. Survey answers obtained in the whole study group are shown in Table 2. Survey answers specific for the cases group are shown in Table 3. This shows the two most and least common answers for each question. Unless otherwise stated statistical analysis by sex, age, and disease category was not statistically different.

**Table 1:** Demographic and clinical characteristics of the study population

Characteristic	Study population		T2DM		COPD		RA		HT	
	Cases	Controls	Cases	Controls	Cases	Controls	Cases	Controls	Cases	Controls
	*n* = 604	n = 604	*n* = 151	*n* = 151	*n* = 151	*n* = 151	*n* = 151	*n* = 151	*n* = 151	*n* = 151
Age, mean ± SD (years)	54.9 ± 13.9	47.1 ± 15.5^a^	52.2 ± 12.8	46.6 ± 15.7^a^	60.3 ± 14.2	53.1 ± 16.7^a^	50.1 ± 13.4	41.6 ± 15.3^a^	56.8 ± 13.1	47 ± 12.1^a^
Age group, *n* (%)										
< 50 years	216 (35.8)	320 (53.0)^a^	68 (45.0)	83 (55.0)	31 (20.5)	57 (37.7)^a^	71 (47.0)	103 (68.2)^a^	46 (30.5)	77 (51.0)^a^
> 50 years	388 (64.2)	284 (47.0)	83 (55.0)	68 (45.0)	120 (79.5)	94 (62.3)	80 (53.0)	48 (31.8)	105 (69.5)	74 (49.0)
Sex, *n* (%)										
Female	377 (62.4)	394 (65.2)	101 (66.9)	95 (62.9)	69 (45.7)	92 (60.9)^b^	126 (83.4)	110 (72.8)^b^	81 (53.6)	97 (64.2)
Male	227 (37.6)	210 (34.8)	50 (33.1)	56 (37.1)	82 (54.3)	59 (39.1)	25 (16.6)	41 (27.2)	70 (46.4)	54 (35.8)
Years of education, *n* (%)										
< 9 years	363 (60.1)	341 (56.5)	90 (59.6)	82 (54.3)	89 (58.9)	76 (50.3)	90 (59.6)	85 (56.3)	94 (62.3)	98 (64.9)
> 9 years	241 (39.9)	263 (43.5)	61 (40.4)	69 (45.7)	62 (41.1)	75 (49.7)	61 (40.4)	66 (43.7)	57 (37.7)	53 (35.1)
Health care, *n* (%)										
Yes	476 (78.8)	453 (75)	122 (80.8)	118 (78.1)	116 (76.8)	121 (80.1)	112 (74.2)	116 (76.8)	126 (83.4)	98 (64.9)^a^
No	128 (21.2)	151 (25)	29 (19.2)	33 (21.9)	35 (23.2)	30 (19.9)	39 (25.8)	35 (23.2)	25 (16.6)	53 (35.1)
Previous clinical trial participation (case group only), *n* (%)										
One	395 (65.4)		89 (58.9)		89 (58.9)		119 (78.8)		98 (64.9)	
Two to three	196 (32.5)		60 (39.7)		56 (37.1)		28 (18.5)		52 (34.4)	
Three to six	10 (1.7)		2 (1.3)		5 (3.3)		2 (1.3)		1 (0.7)	
More than six	3 (0.5)		0 (0)		1 (0.7)		2 (1.3)		0 (0)	

^a^*P* ≤ 0.001; ^b^*P* ≤ 0.05

*T2DM* Type 2 diabetes mellitus, *COPD* chronic obstructive pulmonary disease, *RA* rheumatoid arthritis, *HT* hypertension

**Table 2:** Comparison of the answers between cases and controls^a^

Questions and answers	Cases	Controls	*P* value
	*n* = 604	*n* = 604	
Ethics Committee			
1. What is your opinion about the Ethics Committees?			
I think most of the time they are aware of what is happening in these studies	305 (50.5)	204 (33.8)	< 0.001
I am aware they exist but I do not know their function	133 (22)	165 (27.3)	
I do not know what an Ethics Committee is	111 (18.4)	127 (21)	
I am aware that they exist but I am not sure if they fulfill their functions	45 (7.5)	56 (9.3)	
I think most of the time they are not aware of what is happening in these studies	4 (0.7)	27 (4.5)	
I am sure that most of the time they are not responsible of fulfilling their function	5 (0.8)	24 (4)	
Other opinion	1 (0.2)	1 (0.2)	
2. Which is the main function of an Ethics Committee?			
To ensure that research physicians and their collaborators are adequately qualified	180 (29.8)	192 (31.8)	0.293
I do not know precisely what responsibilities they must fulfill	175 (29)	189 (31.3)	
To review whether the research study characteristics provide benefits to the participants	87 (14.4)	63 (10.4)	
To approve or deny permission to execute a research study	67 (11.1)	62 (10.3)	
To register the experimental drug’s adverse reactions as they occur	59 (9.8)	54 (8.9)	
To interrupt the research study in case they consider that participants could be at risk	30 (5)	39 (6.5)	
To make sure that the informed consent documents are easy to understand	4 (0.7)	5 (0.8)	
Other function	2 (0.3)	0 (0)	
Research studies’ medical care			
3. What is your opinion about the research physicians that participate in pharmaceutical industry-sponsored studies?			
They must be qualified physicians evaluated by international pharmaceutical companies	545 (91.3)	374 (62.1)	< 0.001
I have no opinion on the matter because I do not know any research physician	25 (4.2)	109 (18.1)	
They are like any other physician, the only difference is that they do this activity	20 (3.4)	67 (11.1)	
I believe that most of them participate in this kind of studies only because of the money they are paid	5 (0.8)	41 (6.8)	
I think that they must not be very good physicians	2 (0.3)	10 (1.7)	
Other opinion	0 (0)	1 (0.2)	
4. What is your opinion about the medical care that pharmaceutical industry research studies’ participants receive?			
It is much better than private medical care	464 (77.2)	230 (38.2)	< 0.001
It must be the same as private medical care	81 (13.5)	157 (26.1)	
I do not know if it is better or worse than private medical care	14 (2.3)	128 (21.3)	
I believe the most important fact is that the medical care is free of charge	33 (5.5)	48 (8)	
It is inferior to private medical care	9 (1.5)	36 (6)	
Other reason	0 (0)	3 (0.5)	
5. Do you consider that participating in a clinical trial is an adequate way for patients to get medical care			
Yes	599 (99.2)	537 (88.9)	< 0.001
No	5 (0.8)	67 (11.1)	
6. Why do you believe that participating in a clinical trial is an adequate way to get medical care?			
Because participants receive closer follow-up than with any other medical center	308 (51.6)	175 (32.7)	< 0.001
Because participants receive new treatments that offer more advantages than any other medication available	71 (11.9)	107 (20)	
Because international scientists are monitoring the medication’s effects	93 (15.6)	79 (14.8)	
Because these are very strict studies where all medical adverse reactions are monitored	31 (5.2)	47 (8.8)	
Because the physician is available and can be found easily	53 (8.9)	23 (4.3)	
Although it is not ideal, it is the best way to get medical care if you lack economical resources to pay for it	12 (2)	51 (9.5)	
Because they perform a lot of laboratory tests free of charge	19 (3.2)	34 (6.4)	
Because physicians that do medical research are more qualified	10 (1.7)	19 (3.6)	
7. Why do you believe that participating in a clinical trial is not an adequate way to get medical care?			
Because of potential risks that are not present when using authorized treatments	0 (0)	30 (44.8)	0.005
Because a lot of unnecessary laboratory tests are performed	1 (16.7)	16 (23.9)	
Because treatments are interrupted once the research study is over	3 (50)	13 (19.4)	
Because they require you to assist to a lot of unnecessary consults	1 (16.7)	7 (10.4)	
Did not answered	1 (16.7)	0 (0)	
Other reason	0 (0)	1 (1.5)	
Economical Reimbursement			
8. Do you believe that you must receive an economical reimbursement (money) for participating in a research study?			
Yes	299 (49.5)	321 (53.1)	0.205
No	305 (50.5)	283 (46.9)	
9. What is your opinion about receiving an economical reimbursement (money) for participating in a research study?			
There should not be monetary supportive stimuli unless they are strictly justified	274 (45.4)	223 (36.9)	< 0.001
That it is necessary in order to cover the costs of extra transportation that participating in a study implies	240 (39.7)	183 (30.3)	
I think it is payment for risking my health	24 (4)	73 (12.1)	
Other reason	17 (2.8)	36 (6)	
I think it is a good payment for participating in a research study	13 (2.2)	39 (6.5)	
I think it is used to convince some people to enroll in a research study	13 (2.2)	36 (6)	
Monetary supportive stimuli are not proportionate to the potential risks that participating in a research study implies	23 (3.8)	14 (2.3)	

^a^Data are given as *n* (%)

**Table 3:** Comparison of survey questions only for cases by disease group^a^

Questions and answers		Disease Group				
	Total	T2DM	COPD	RA	HT	*P v*alue
	(*n* = 604)	(*n* = 151)	(*n* = 151)	(*n* = 151)	(*n* = 151)	
Enrollment						
10. How were you invited to participate in your current or most recent research study?						
By a clinical researcher participating in the study.	190 (31.5)	43 (28.5)	51 (33.8)	76 (50.3)	20 (13.2)	< 0.001
In the research physician’s private consult.	120 (19.9)	25 (16.6)	36 (23.8)	26 (17.2)	33 (21.9)	
A patient who participates in the research study invited me.	115 (19)	52 (34.4)	18 (11.9)	14 (9.3)	31 (20.5)	
Other media.	65 (10.8)	14 (9.3)	14 (9.3)	9 (6)	28 (18.5)	
In a disease detection campaign.	45 (7.5)	9 (6)	12 (7.9)	6 (4)	18 (11.9)	
In a private medical consult.	39 (6.5)	5 (3.3)	9 (6)	6 (4)	19 (12.6)	
Notice published at a hospital.	16 (2.6)	2 (1.3)	5 (3.3)	7 (4.6)	2 (1.3)	
Newspaper advertisement.	13 (2.2)	1 (0.7)	5 (3.3)	7 (4.6)	0 (0)	
Radio advertisement.	1 (0.2)	0 (0)	1 (0.7)	0 (0)	0 (0)	
Notice published online.	0 (0)	0 (0)	0 (0)	0 (0)	0 (0)	
Television advertisement.	0 (0)	0 (0)	0 (0)	0 (0)	0 (0)	
11. Why did you decided to participate in your current research study?						
Because this is the best way to get medical care.	222 (36.8)	54 (35.8)	54 (35.8)	56 (37.1)	58 (38.4)	< 0.001
Because the physician’s care is more cautious.	159 (26.3)	52 (34.4)	41 (27.2)	31 (20.5)	35 (23.2)	
Because I have not responded to available treatments.	112 (18.5)	21 (13.9)	15 (9.9)	28 (18.5)	48 (31.8)	
Because my participation aids scientific medical progress.	65 (10.8)	11 (7.3)	25 (16.6)	22 (14.6)	7 (4.6)	
Because I have no other way to get clinical care.	23 (3.8)	5 (3.3)	8 (5.3)	10 (6.6)	0 (0)	
Because everything is free.	19 (3.1)	7 (4.6)	7 (4.6)	2 (1.3)	3 (2)	
Other reason.	4 (0.7)	1 (0.7)	1 (0.7)	2 (1.3)	0 (0)	
Informed Consent						
12. Have you fully read the informed consent letter required to accept enrolling in the research study?						
Yes	546 (90.4)	138 (91.4)	137 (90.7)	129 (85.4)	142 (94)	0.079
No	58 (9.6)	13 (8.6)	14 (9.3)	22 (14.6)	9 (6)	
13. Why did you not read the informed consent letter?						
Because I fully trust that my physician would not put my health at risk.	33 (56.9)	4 (30.8)	9 (64.3)	14 (63.6)	6 (66.7)	0.115
Because I did not have enough time to read it.	11 (19)	6 (46.2)	2 (14.3)	2 (9.1)	1 (11.1)	
Because the document was very long.	8 (13.8)	2 (15.4)	3 (21.4)	1 (4.5)	2 (22.2)	
Other reason.	3 (5.2)	1 (7.7)	0 (0)	2 (9.1)	0 (0)	
Because I did not understand it, the text was not clear.	3 (5.2)	0 (0)	0 (0)	3 (13.6)	0 (0)	
14. How much time did the research physician take to explain the research study’s process, risks and benefits, as well as your rights and obligations as a participant?						
More than 30 min.	262 (43.4)	56 (37.1)	33 (21.9)	64 (42.4)	109 (72.2)	< 0.001
20 to 30 min.	130 (21.5)	50 (33.1)	33 (21.9)	25 (16.6)	22 (14.6)	
10 to 20 min.	107 (17.7)	27 (17.9)	33 (21.9)	35 (23.2)	12 (7.9)	
No more than 10 min.	97 (16.1)	17 (11.3)	46 (30.5)	26 (17.2)	8 (5.3)	
Less than 5 min.	8 (1.3)	1 (0.7)	6 (4)	1 (0.7)	0 (0)	
15. How satisfied are you with the explanation received about the research study’s process, risks and benefits, as well as the protection you have in case of an adverse reaction related to the experimental drug?						
Very satisfied.	326 (54)	81 (53.6)	78 (51.7)	70 (46.4)	97 (64.2)	0.043
Satisfied.	249 (41.2)	67 (44.4)	63 (41.7)	68 (45)	51 (33.8)	
Neither satisfied nor unsatisfied.	20 (3.3)	3 (2)	6 (4)	10 (6.6)	1 (0.7)	
Unsatisfied.	7 (1.2)	0 (0)	3 (2)	2 (1.3)	2 (1.3)	
Very unsatisfied.	2 (0.3)	0 (0)	1 (0.7)	1 (0.7)	0 (0)	
Cases’ experience participating in an industry-sponsored research trial						
16. How satisfied are you with your participation in this research study?						
Very satisfied.	376 (62.3)	90 (59.6)	89 (58.9)	83 (55)	114 (75.5)	0.009
Satisfied.	215 (35.6)	61 (40.4)	58 (38.4)	61 (40.4)	35 (23.2)	
Neither satisfied nor unsatisfied.	6 (1)	0 (0)	1 (0.7)	4 (2.6)	1 (0.7)	
Unsatisfied.	3 (0.5)	0 (0)	1 (0.7)	2 (1.3)	0 (0)	
Very unsatisfied.	4 (0.7)	0 (0)	2 (1.3)	1 (0.7)	1 (0.7)	
17. How satisfied are you with the time your research physician dedicates you?						
Very satisfied.	413 (68.4)	106 (70.2)	94 (62.3)	90 (59.6)	123 (81.5)	0.003
Satisfied.	179 (29.6)	44 (29.1)	53 (35.1)	57 (37.7)	25 (16.6)	
Neither satisfied nor unsatisfied.	5 (0.8)	1 (0.7)	1 (0.7)	2 (1.3)	1 (0.7)	
Unsatisfied.	3 (0.5)	0 (0)	0 (0)	1 (0.7)	2 (1.3)	
Very unsatisfied.	4 (0.7)	0 (0)	3 (2)	1 (0.7)	0 (0)	
18. How satisfied are you with your research physician’s sense of humanity?						
Very satisfied.	466 (77.2)	124 (82.1)	113 (74.8)	103 (68.2)	126 (83.4)	0.019
Satisfied.	133 (22)	27 (17.9)	36 (23.8)	47 (31.1)	23 (15.2)	
Neither satisfied nor unsatisfied.	1 (0.2)	0 (0)	1 (0.7)	0 (0)	0 (0)	
Unsatisfied.	2 (0.3)	0 (0)	0 (0)	0 (0)	2 (1.3)	
Very unsatisfied.	2 (0.3)	0 (0)	1 (0.7)	1 (0.7)	0 (0)	
19. How easy is to talk to your research physician? (Availability)						
Very easy.	447 (74)	114 (75.5)	108 (71.5)	97 (64.2)	128 (84.8)	0.021
Easy.	143 (23.7)	36 (23.8)	37 (24.5)	49 (32.5)	21 (13.9)	
Neither easy nor difficult.	10 (1.7)	1 (0.7)	5 (3.3)	3 (2)	1 (0.7)	
Difficult.	2 (0.3)	0 (0)	0 (0)	1 (0.7)	1 (0.7)	
Very difficult.	2 (0.3)	0 (0)	1 (0.7)	1 (0.7)	0 (0)	
20. How satisfied are you when talking to the research physician?						
Very satisfied.	457 (75.7)	117 (77.5)	105 (69.5)	106 (70.2)	129 (85.4)	0.019
Satisfied.	136 (22.5)	34 (22.5)	40 (26.5)	41 (27.2)	21 (13.9)	
Neither satisfied nor unsatisfied.	5 (0.8)	0 (0)	2 (1.3)	3 (2)	0 (0)	
Very unsatisfied.	4 (0.7)	0 (0)	3 (2)	1 (0.7)	0 (0)	
Unsatisfied.	2 (0.3)	0 (0)	1 (0.7)	0 (0)	1 (0.7)	
21. How satisfied are you with the results obtained regarding the research medical care of your disease?						
Very satisfied.	444 (73.5)	110 (72.8)	104 (68.9)	103 (68.2)	127 (84.1)	0.055
Satisfied.	144 (23.8)	36 (23.8)	41 (27.2)	43 (28.5)	24 (15.9)	
Neither satisfied nor unsatisfied.	12 (2)	5 (3.3)	4 (2.6)	3 (2)	0 (0)	
Very unsatisfied.	3 (0.5)	0 (0)	2 (1.3)	1 (0.7)	0 (0)	
Unsatisfied.	1 (0.2)	0 (0)	0 (0)	1 (0.7)	0 (0)	

^a^Data are given as *n* (%)

*T2DM* Type 2 diabetes mellitus, *COPD* chronic obstructive pulmonary disease, *RA* rheumatoid arthritis, *HT* hypertension

### Survey questions for cases and controls

Cases significantly answered that ethics committees (EC) are aware of what is happening in the studies (50.5% vs. 33.8%, *P* = < 0.001), that research physicians are qualified and evaluated by international pharmaceutical companies (90.0% vs. 62.1%, *P* = 0.001), that medical care of the ISRT is much better than their usual medical care (77.2% vs. 38.2%, *P* ≤ 0.001), that participating in a clinical trial is an adequate way to receive medical care for their disease (99.2% vs. 88.9%, *P* = 0.001); the reason was that participants get closer medical care (51.6% vs. 32.7%, *P* = < 0.001), but the main inconvenience was that treatments are interrupted once the research is concluded (50% vs. 19.4%, *P* = 0.005). They also answered that economical reimbursement was an acceptable supportive stimulus only when it is justified (45.5% vs. 36.9%, *P* ≤ 0.001) or necessary to cover extra transportation costs (39.7% vs. 30.3%, *P* ≤ 0.001).

Controls when contrasted to cases, significantly answered that a major proportion of physicians participate in these studies because of the money they are paid (6.8% vs. 0.8%, *P* ≤ 0.001), that medical care of the ISRT is inferior to typical medical care (6.0% vs. 1.5%, *P* ≤ 0.001). They also thought that participating was an adequate way to get medical care because they receive new treatment options (20.0% vs. 11.9%, *P* ≤ 0.001), that it was unreasonable because of the risks of experimental drugs compared to non-investigational treatments (44.8% vs. 0%, *P* = 0.005), and that they must receive an economical reimbursement as a compensation for risking their health (12.1% vs. 4%, *P* ≤ 0.001), and a fair payment for participating in a research study (6.5% vs. 2.2%, *P* ≤ 0.001).

There was no difference between cases and controls regarding the main function of the EC (to ensure the qualification of research physician and collaborators, 29.8% vs. 31.8%, *P* = 0.293), and that they must receive economical reimbursement for participating in a research study (49.5% vs. 53.1%, *P* = 0.205). However, a higher proportion of controls thought that EC were not aware of what happens in clinical trials (cases 0.7% vs. controls 4.5%, *P* ≤ 0.001) or that they were not responsibly fulfilling their function (cases 0.8% vs. controls 4%, *P* ≤ 0.001).

### Survey questions only for cases

Cases were invited to pharmaceutical research studies mainly by a clinical researcher involved in the study (51.4%), and by other study participants (19%). They decided to participate because it is the best way to get medical care (36.8%), followed by “physician care of the patient is more cautious” (26.3%). The least given answer was because everything is free (3.1%). The IC of the pharmaceutical clinical trial was fully and deeply read by 90.4% of the cases. Those participants who did not fully read the IC (9.6%) expressed that they skipped reading some parts of the IC letter because they trusted their physicians (56.9%), followed by not having enough time to read it (19%). The researcher explained the protocol, risks, and benefits as well as their rights and obligations as a participant most of the times in more than 30 min (43.4%). Nevertheless, in some cases this time was reduced to 10 min (16.1%) or less (1.3%). Regarding satisfaction, most cases were satisfied or very satisfied with their overall study participation (35.6 and 62.3%, respectively), the time research physicians took to explain the research study (41.2 and 54%, respectively), the researcher or staff availability (23.7 and 74%, respectively) the sense of humanity of the research physician (22 and 77.2%, respectively), and the outcomes obtained through the medical care received (23.8 and 73.5% respectively).

## Discussion

In this multicenter, cross-sectional, comparative survey study conducted in a developing country, participants of ISRT were more aware than controls of the responsibility and function of the EC in pharmaceutical industry trials, the higher quality of their medical services and the researcher’s physicians, the protection by the EC, safety and overall satisfaction during protocol participation, and the reasons behind economical reimbursement.

More than half of the ISRT conducted in low- and low-middle income countries focus on investigational drugs for chronic diseases [6]. Although several studies have assessed ISRT in developing countries, they focus on assessing patients who are frequently non-responders to standard-of-care medications, and consequently, forced to explore investigational drugs (e.g., cancer, type C hepatitis) [12, 13]. A strength of our study is that our surveyed participants had T2DM, HT, COPD and RA, very common disorders with many accessible therapeutic choices and who decided to be recruited into clinical trials despite all these good available medications for their diseases. Therefore, their responses might better reflect the perception of their participation in pharmaceutical ISRT. A recent study by Wu E. et al. of 1200 participants with hepatitis C from the United States and urban and rural China, assessed the perspectives and concerns of research participants and non-participants about ISRT [12]. They found that while US participants were concerned about safety, privacy, and confidentiality, Chinese had concerns about self-benefit, free medical care, and economical reimbursement [12]. These findings show that sociocultural and economic factors have a huge influence on how people see clinical research. A previous report of the first section of the present survey showed that participation in this type of studies improves their future perception [8]. Other authors have observed this finding [5, 12, 14, 15]. Moreover, Kost et al. in 2014 assessed the experiences of participants in NIH-supported clinical research centers. They distributed almost 19,000 surveys obtained response from 29% finding that 73% top-rated their overall research experience and up to 63% would recommend participating in a clinical trial [14]. Likewise, the 2017 CISCRP’s Perceptions & Insights Study assessed more than 12,000 patients, mostly previous participants of clinical trials (82%) with diverse medical conditions via an online international survey. They reported that 54% would definitely recommend participating in clinical research and that 33% are somewhat willing and 59% are very willing to participate in another study [15]. These studies disclose the shared similarities and notable differences among research participants’ perspectives worldwide.

Ethical oversight has been a concern since the beginning of the globalization in pharmaceutical clinical research and, although ethical compliance has improved over the last decade, areas of opportunities remain to be revised [1, 9, 11, 16]. The majority of our study population had an overall positive perception about EC. Nevertheless, almost one in five cases and controls did not know what an EC was, as well as their functions and responsibilities. Current and potential participants seems to lack awareness of the safety monitoring that is entailed in clinical trials [17]. Similarly, when Wu et al. asked their study population to rank how relevant it was for them to be informed that a study received EC review before the research starter visit, 95% of US patients considered it important or very important, compared to less than 80% in rural and urban Chinese [12]. This finding highlights the sense of safety and validity that EC represent to patients who are aware of their key role for trial conduction.

Mandava et al. reported that the educational level of participants or living in a developed or developing country does not seem to affect their research trial comprehension [18]. Moreover, a key factor associated to better protocol understanding of IC letter is the amount of time designated to explain the study protocol [19]. In our study, most of the time, more than 20–30 min were designated for explanation to each participant; this could possibly explain their high level of satisfaction with the information received, the amount of time researchers dedicated to them as well as the sense of humanity and availability of the researchers. Similar to the outpatient clinic scenario, translating the shared decision-making approach to research trials promotes trust and empowers the choice of the patient for accepting or declining to participate [20].

In this study, most controls and virtually all cases considered that participating in a clinical trial is an adequate way for patients to get medical care for their condition, mainly because of the closer follow-up they receive. Cox and McDonald conducted in-depth interviews of previous research participants in order to obtain their narratives of being participants of clinical trials and clustered the results into four categories: “surviving”, “being conscripted”, “being a good citizen” and “health consuming” [21]. The narrative of our study population could be categorized as “health consuming”: patients with a chronic illness that has treatment options that could be managed in a more effective way through research, even perceiving trials as part of their standard care [21] This scenario could be more prevalent in years to come. To date, it is undeniable the considerable amount of patients worldwide who lack access to qualified health services [22]. Participants from this and others studies have stated that research trials enables them to get access to otherwise unreachable medical services [12]. Therefore, even if it is not their goal, ISRT improve the health conditions of some untreated patients through their higher-quality medical care.

Both cases and controls were split when asked whether or not economical reimbursement was a must for them to participate in a research study. Patients often view economical reimbursement as a sign of appreciation for the time spent attending the appointments of their research trial, which did not “blind” them on the risks that implies participating in an ISRT [23, 24]. On the other hand, members of Institutional Review Boards disapprove economical reimbursements because of their potential use as an undue incentive to enroll patients. Interestingly, most of our study population expressed a more balanced opinion, stating that all monetary supportive stimuli have to be strictly justified. As suggested by Wertheimer, economic reimbursements should be individualized and established for each study based on a risk-benefit evaluation, in order to show respect for the autonomy of the participants [25]. Therefore, it could be argued that it is ethically correct and beneficial to pursue the implementation of fair economical reimbursements while designing clinical trials, even in the setting of developing countries.

Regarding participants’ satisfaction, most cases in our study expressed that they were satisfied or very satisfied with their overall participation (97.9%) and with the results obtained (97.3%). These results strongly contrasts with the opinions of research participants in developed countries. Kost et al., reported 75% of participants top-rated their overall experience [14]; in the 2015 CISCRP survey, less than half of the participants considered that their clinical research study greatly exceeded (19%) or exceeded their expectations (27%) [5]. Although more studies are needed, it seems that research participants of ISRT conducted in low- and low-middle income countries more positively perceive and are more satisfied with their role as participants and the overall experience of participating in a clinical trial than their peers from developed countries.

This study has several limitations. First, the trail design does not permit to study the influence that participating in a clinical trials have directly on patients’ perceptions and opinions about clinical trials and only associations can be made. Future studies that assess patients’ perception before and after their first participation in a clinical trial could clarify this issue. Second, although the study was validated in a pilot study, there are still chance of misunderstanding, or skipping overall the whole survey. Nevertheless, the majority of surveys were answered with a staff member around in order to clarify questions and ensure the survey were correctly and fully answered. Third, study sample was not calculated, instead we draw a convenience sample. Nevertheless, we considered our population to be large enough in order to reduce the possibility of a sampling error to occur. Fourth, although, the majority of the cases in our study were current participating in their first clinical trials and were blind to treatment, cases that were at their second or further clinical trial were not assessed for previous exposure to placebo or active substance. The influence that had been under placebo vs active substance may have on participants’ perceptions and opinions about research trials is a strong point to be assessed which future studies must approach. Fifth, because cases must have attended at least their sixth visit in order to be included in the study, some questions about process that occur early in the study (e.g. informed consent, enrollment, etc.) could carry a memory bias. Nevertheless, we considered that being at their six visit was an appropriate time to have gained experience in what participating in a clinical trial really means, justifying not asking cases right away after their enrollment in clinical trials.

## Conclusion

Previous and current participants of ISRT have a more positive attitude towards ethics committees, the quality of medical care of the research trials, and the main purpose of economical reimbursements than non-participants. The gratifying experiences of participants in previous or current clinical trials may be accountable for this. In both groups, however, there is still a wide spectrum of opportunities to improve the perception about their participation. Special programs addressed to the population in our country and likely in low-middle income countries regarding the right way to conduct recruitment, the IC procedure, benefits, autonomy, freedom to participate and to drop out from the study, and other related topics are clearly needed, assessing scientifically the value of each of these programs. Moreover, it is necessary to assess perception of participants in non-professionalized vs. professionalized research sites in a multinational trial.

## Abbreviations

COPD: Chronic obstructive pulmonary disease
EC: Ethics committees
HT: Arterial hypertension
IC: Informed consent
ISRT: Industry-sponsored research trials
RA: Rheumatoid arthritis
T2DM: Type 2 diabetes mellitus
